# Nutritional status and its effect on treatment outcome among HIV infected clients receiving HAART in Ethiopia: a cohort study

**DOI:** 10.1186/s12981-016-0116-9

**Published:** 2016-09-22

**Authors:** Sadikalmahdi Hussen, Tefera Belachew, Nezif Hussien

**Affiliations:** 1 Clinical Pharmacy Department, School of Pharmacy, Jimma University, Jimma, Ethiopia; 2 Human Nutrition Department, Jimma University, Jimma, Ethiopia

**Keywords:** Malnutrition, CD4, Death, Survival, Ethiopia

## Abstract

**Purposes:**

The aim of this study was to determine the effects of nutritional status at the start of highly active anti-retroviral therapy on treatment outcomes among HIV/AIDS patients taking HAART at Jimma University Specialized Hospital.

**Methods:**

We performed a retrospective cohort study involving 340 adults who started highly active anti-retroviral therapy. The patients have been clinically followed for 2 years. Data were extracted from paper based medical charts by trained data collectors from January 30 to February 28, 2014 using data collection format. We entered data into Epi data version 3.1 and then exported to SPSS for windows version 21. Predictors of CD4 change were identified using multivariable linear regression model. Time to an event (death) was estimated by Kaplan–Meier and predictors of mortality were identified by Cox proportional hazard model.

**Results:**

Out of 340 patients, 42 patients died during the follow-up. Twenty-five (59.5 %) deaths were from malnourished group. Age, baseline CD4, sex, baseline HAART and marital status were significant predictors of immunologic recovery at different time points. Malnutrition was associated with lower CD4 recovery and greater hazard of death.

**Conclusions:**

Malnutrition tends to decrease CD4 recovery and predisposes patient to early death.

## Background

Treatment of HIV-infected patients with highly active antiretroviral therapy (ART) leads to immune reconstitution as shown by increases in CD4 lymphocyte counts, decreased risk of opportunistic infections and improved survival [1, 2]. However, all patients do not have an optimal response to therapy. Some patients have slow and incomplete recovery of immune function and remain at greater risk of developing opportunistic infections and death than those who show more rapid immune reconstitution [3]. Patients may die with an undetectable viral load and adequate CD4 count recovery [2]. Therefore, adjunctive treatments that accelerate the recovery of immune function or that address other related causes of mortality may provide additional gains in survival in patients with HIV starting HAART.

Even though, previous studies showed malnutrition was independent predictor of death in patients taking HAART [4–8] in different countries, there were conflicting results on impact of malnutrition at HAART initiation on immunologic recovery at different time periods after HAART initiation, some studies showed malnutrition does not prevent an excellent response to HAART [9] while, other suggest poor immunological response [10].

However, no previous study had holistically examined the impact on survival, CD4 recovery and occurrence of opportunistic infections of malnutrition at the time of starting HAART. Furthermore, there were few studies in Africa and no study done in Ethiopia that examined effect of malnutrition at the initiation of HAART on treatment outcome. It is possible that malnutrition may impair the immune response to HAART, prolong the period during which patients are at risk of opportunistic infection and directly or indirectly increasing the risk of death. Malnutrition may therefore, represent a potentially reversible cause of increased mortality in patients who are initiating ART.

## Methods

### Study design and participants

We conducted retrospective cohort study at Jimma University Specialized Hospital, the only teaching and referral hospital with bed capacity of 450 in the South Western part of the country providing specialized health service for approximately 9000 inpatients and 80,000 outpatients each. The ART clinic of the hospital started providing service to people living with HIV/AIDS (PLWA) in 2002. Since establishment the clinic had 3700 patients following care and treatment [11]. The primary data was collected from September 11, 2006 to September 10, 2011. Data was extracted from the medical record from January 30 to February 28, 2014. The sample size was calculated by single proportion formula used for cohort studies, which assumes proportion of mortality in malnourished group to be 61.8 % and proportion of mortality in well-nourished group to be 46.8 with 95 % confidence interval, 80 % power and 1:1 ratio of unexposed versus exposed. The sample size calculated was 340 patients; one hundred seventy (170) patients in both malnourished and well-nourished groups. The medical records of adult patients who started HAART between September 2006 and September 2011 were isolated. The isolated medical charts were categorized into malnourished and well-nourished groups based on their BMI at the start of HAART. Malnutrition was defined as a BMI <18.5, while BMI ≥18.5 was defined as a well-nourished as per WHO criteria. All patients whose age was greater than 14 were included in the study. Pregnant women’s (BMI and nutrient metabolism vary during pregnancy), patients with incomplete data on weight, height and outcome variables, transferred-out during follow-up were excluded from the study. The data were collected by clinically trained data collectors using data collection tool adapted from national ART clinic intake form, ART follow up form and anti-retroviral drugs and patient information sheet. The endpoint of this study was death. The patients were followed until the occurrence of event (death) or until 2 years (end of study). Patients who were alive at the end of study were censored. The survival time was calculated in days using date of starting treatment and date of an event or date censored.

### Statistical methods

The data was cleaned, edited and completed using Epi data version 3.1 and exported to SPSS for window version 21 for analysis. The main analysis in this study was linear regression for CD4 recovery and survival analysis for death. Variables with p value of <0.25 in bivariate analysis and BMI were fitted to multivariable cox-proportional analysis model to identify independent predictors of CD4 change at all time points. Kaplan–Meier survival analysis was done to estimate the survival time. Log-rank test was used to compare the KM curves for two or more categories of patients on HAART. Predictors of event (death) over a period of time t, was analyzed by Cox proportional hazard model. The level of significance was set at p value less than 0.05 and 95 % confidence intervals (CI) were used throughout. Multi-collinerity was checked for independent variables before fitting into multivariable analysis.

## Results and discussion

### Results

#### Baseline characteristics

We followed all patients for 2 years. There were a total of 42 deaths during the follow-up period. Twenty-five (59.5 %) deaths were from malnourished group. The mean age of the study participants was 34 [IQR 26–38] years. The median age was 30 years and 200 (58.8 %) patients were females. Mean baseline BMI was 1.50 [IQR 1.0–2.00] kg/m^2^. The median baseline CD4 count was 144.5 [IQR 89–209] cells/mm^3^ (Tables 1, 2).

**Table 1:** Baseline demographic characteristics and associated mortality and opportunistic infections of 340 HIV-infected patients in Jimma University Specialized Hospital, from January 2006 to December 2011

Characteristics	Number of patients (%)	Number of deaths (%)	Number of OI (%)
Sex			
Male	140 (41.2)	19 (45.2)	38 (45.8)
Female	200 (58.8)	23 (54.8)	45 (54.2)
BMI			
<18.5	170 (50)	25 (59.5)	55 (66.3)
≥18.5	170 (50)	17 (40.5)	28 (33.7)
Religion			
Orthodox	203 (59.7)	29 (69)	44 (53)
Muslim	100 (29.4)	13 (31)	26 (31.3)
Protestant	31 (9.1)	0	11 (13.3)
Catholic	2 (0.6)	0	1 (1.2)
Others	2 (0.6)	0	0
Age (years)			
<30	174 (51.2)	21 (50.0)	46 (55.4)
30–39	94 (27.6)	11 (26.2)	21 (25.3)
40–49	57 (16.8)	6 (14.3)	12 (14.5)
>50	15 (4.4)	4 (9.5)	4 (4.8)
Marital status			
Single	43 (12.6)	7 (16.7)	10 (12.0)
Married	116 (34.1)	16 (38.1)	36 (43.4)
Widowed	22 (6.5)	1 (2.4)	5 (6.0)
Divorced	44 (12.9)	8 (19.0)	9 (10.8)
Occupation			
Gov’t employee	98 (28.8)	14 (33.3)	19 (22.9)
Merchant	5 (1.5)	0 (0)	2 (2.4)
Unemployed	189 (55.6)	25 (59.5)	51 (61.4)
Private org	37 (10.9)	2 (4.8)	10 (12.0)
NGO’s	5 (1.5)	1 (2.4)	0 (0)
Educational status			
Not educated	69 (20.3)	4 (9.5)	24 (28.9)
Primary	122 (35.9)	17 (40.5)	25 (30.1)
Secondary	106 (31.2)	14 (33.3)	21 (25.3)
Tertiary	42 (12.4)	7 (1.7)	12 (14.4)

**Table 2:** Baseline clinical characteristics and associated mortality and opportunistic infections of HIV-infected patients in Jimma University Specialized Hospital, between January 2006 and December 2011

Characteristics	Number of patients (%)	Number of deaths (%)	Number of OI (%)
WHO clinical stage			
Stage I	65 (19.1)	6 (14.3)	15 (18.1)
Stage II	95 (27.9)	9 (21.4)	15 (18.1)
Stage III	144 (42.4)	17 (40.5)	44 (53.0)
Stage IV	36 (10.6)	10 (23.8)	9 (10.8)
HAART regimen			
Stavudine based	219 (64.4)	19 (45.2)	60 (72.3)
Zidovudine based	72 (21.2)	12 (28.6)	18 (21.7)
Tenofovir based	49 (14.4)	11 (26.2)	5 (6.0)
Base line CD4			
>350	5 (1.5)	0 (0.0)	2 (2.4)
200–350	94 (27.6)	7 (16.7)	19 (22.9)
100–199	139 (40.9)	15 (35.7)	37 (44.6)
<100	102 (30.0)	20 (47.6)	25 (30.1)
Risky behaviour			
Yes	120 (35.3)	14 (33.3)	58 (69.9)
No	220 (64.7)	28 (67.7)	25 (30.1)
Cotrimoxazole prophylaxis			
Yes	330 (97.1)	42 (100.0)	81 (97.6)
No	10 (2.9)	0 (0.0)	2 (2.4)
Fluconazole prophylaxis			
Yes	20 (5.9)	8 (19.1)	5 (6.0)
No	320 (94.1)	34 (80.9)	78 (94.0)
INH prophylaxis			
Yes	62 (18.2)	7 (16.7)	20 (24.1)
No	278 (81.8)	35 (83.3)	63 (75.9)

#### Immunologic recovery

##### Immunologic recovery at 6 months

Age was the only independent predictor of immunologic recovery. For 1 year increase in the age of the patient, CD4 decreases by 3.40 cells/mm^3^ (p = 0.003). CD4 count of malnourished group decreases by 12.40 cells/mm^3^ (p = 0.500) compared to well-nourished group (Table 3).

**Table 3:** Multivariate predictors of CD4 change at 6 months in HIV-infected patients at Jimma University Specialized Hospital, January 2006 to December 2011

Variables	Number	B [95 % CI]	p value
Age	340	−3.4 [−5.5, −1.2]	0.003
Marital status			
Single	43	13.7 [−61.7, 89.2]	0.720
Married	116	57.2 [−4.2, 118.7]	0.068
Divorced	44	3.5 [−66.1, 73.1]	0.922
Widowed	22	Reference	
BMI			
<18.5	170	−12.4 [−48.8, 23.9]	0.5
≥18.5	170	Reference	
Baseline co morbidity			
Yes	20	25.6 [−40.3, 91.4]	0.445
No	320	Reference	
CD4 count			
<100	5	126.9 [−13.8, 267.6]	0.077
100–199	94	150.1 [10.2, 290.0]	0.036
200–350	139	107.2 [−34.3, 248.7]	0.137
>350	102	References	

##### Immunologic recovery at 12 months

Age and baseline CD4 count were independent predictors of immunologic recovery. For 1 year increase in the age of the patient CD4 decreases by 1.91 cells/mm^3^ (p = 0.005). CD4 count of patients with baseline CD4 count in the range of 100–199 increases by 201.29 cells/mm^3^ (p = 0.047) compared to patients with baseline CD4 count greater than 350. CD4 count of malnourished patients decreases by 21.5 cells/mm^3^ (p = 0.321) compared with well-nourished group at 12 months (Table 4).

**Table 4:** Multivariate predictors of CD4 change at 12 months in HIV-infected individuals at Jimma University Specialized Hospital, January 2006 to December 2011

Variables	Numbers	B [95 % CI]	p value
Age	340	−1.91 [−3.238, −0.582]	0.005
Sex			
Female	200	Reference	
Male	140	−38.67 [−81.973, 4.633]	0.080
BMI			
<18.5	170	−21.54 [−64.197, 21.109]	0.321
≥18.5	170	Reference	
HAART regimen			
Stavudine based	219	25.57 [−34.277, 85.420]	0.401
Zidovudine based	72	−17.53 [−87.25, 52.190]	0.621
Tenofovir based	49	Reference	
Baseline CD4			
<100	5	188.75 [−9.859, 387.359]	0.062
100–199	94	201.29 [2.916, 399.661]	0.047
200–350	139	133.33 [−64.785, 81.773]	0.186
>350	102	Reference	
WHO stage			
Stage 1	65	Reference	
Stage 2	95	−15.96 [−78.139, 46.212]	0.614
Stage 3	144	24.17 [−33.426, 81.773]	0.179
Stage 4	36	−63.89 [−157.331, 29.548]	0.409
Educational status			
Not educated	69	Reference	
Primary	122	52.75 [−4.906, 110.408]	0.073
Secondary	106	40.11 [−18.174, 98.400]	0.177
Tertiary	42	40.113 [−76.277, 83.098]	0.933

##### Immunologic recovery at 24 months

In multivariate linear regression analysis age, sex, marital status and baseline HAART were significant predictors of immunologic recovery at 24th month. For 1 year increase in age of the patients, CD4 increases by 2 cells/mm^3^ (p = 0.005). Females had increased CD4 count by 104.3 cells/mm^3^ compared to males. Married patients had 138.56 cells/mm^3^ (p = 0.005) higher CD4 count compared to widowed patients. CD4 count of patients who started treatment with Stavudine based regimen increases by 87.49 cells/mm^3^ (p = 0.036) compared to CD4 count of patients who started with Tenofovir based regimen. Malnourished group has 22.4 cells/mm^3^ lower increases in CD4 count compared to well-nourished group (Table 5).

**Table 5:** Multivariate predictors of CD4 change at 24 months in HIV-infected individuals at Jimma University Specialized Hospital, January 2006 to December 2011

Variables	Numbers of patients	B [95 % CI]	p value
Age	340	−2.11 [−3.556, −0.665]	0.005
Sex			
Female	200	Reference	
Male	140	−104.31 [−170.702, −37.923]	0.002
Marital status			
Single	43	45.10 [−66.086, 156.294]	0.424
Married	116	138.56 [42.449, 234.655]	0.005
Divorced	44	52.47 [−55.935, 160.873]	0.340
Widowed	22	Reference	
Baseline HAART			
Stavudine based	219	87.49 [5.960, 169.015]	0.036
Zidovudine based	72	16.31 [−75.057, 107.678]	0.724
Tenofovir based	49	Reference	
BMI			
<18.5	170	−22.42 [−81.040, 36.202]	0.451
≥18.5	170	Reference	
Educational level			
Not educated	69	−86.18 [−195.893, 23.534]	0.123
Elementary	122	12.63 [−89.442, 114.698]	0.807
Secondary	106	−38.30 [−139.605, 63.006]	0.456
Tertiary	42	Reference	
Base line CD4			
<100	5	83.84 [−127.555, 295.233]	0.434
100–199	94	87.16 [−122.670, 296.992]	0.413
200–350	139	24.14 [−184.264, 232.534]	0.819
>350	102	Reference	

##### Death

**Fig. 1:**
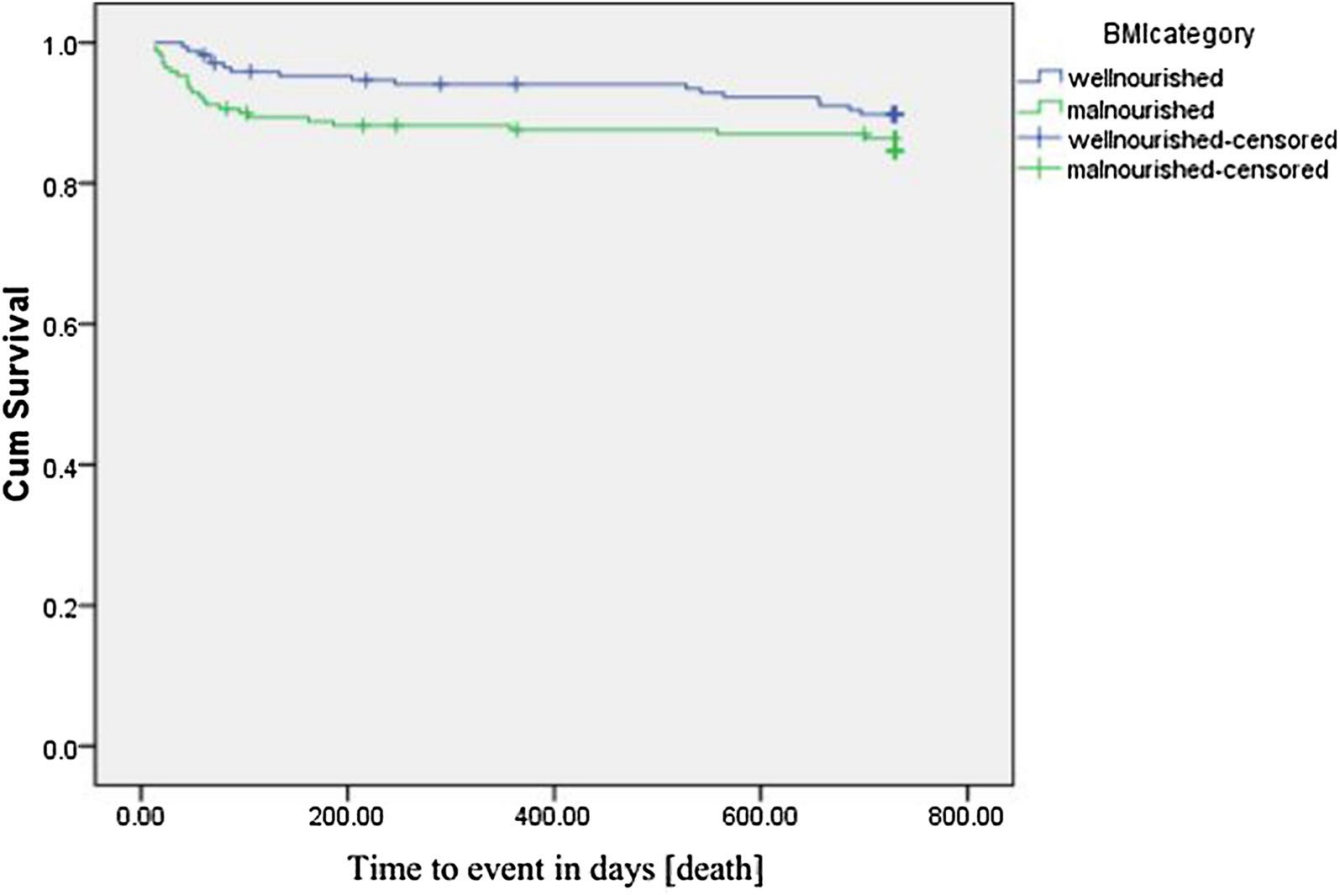
Kaplan–Meier plots of nutritional status in HIV infected individuals in cohort of patients at Jimma University specialized hospital, January 2006 to December 2011

In Kaplan–Meier bivariate cox-proportional analysis survival times significantly differ among groups of baseline CD4 (p = 0.014), WHO clinical stage (p = 0.022), baseline HAART (p = 0.010) and Fluconazole prophylaxis (p = 0.000). Even though, malnourished patients had shorter survival times compared to well-nourished group, it was not statistically significant (p = 0.170) (Fig. 1).

In multivariable cox proportional model four variables were found to be significant predictors of mortality. These were Zidovudine based regimen (AHR = 4.182, 95 % CI 1.550, 11.282, p = 0.005), Tenofovir (AHR = 5.156, p = 0.001), Fluconazole prophylaxis (HR = 5.639, 95 % CI 1.811, 17.563, p = 0.003), age above 50 years (AHR = 4.783, p = 0.040) and CD4 greater than 200 (HR = 0.287, 95 % CI 0.097, 0.848, p = 0.002). Malnourished patients were 1.3 times at risk of death compared to well-nourished group [AHR = 1.460, 95 % CI (0.648, 3.287), p = 0.361] (Table 6).

**Table 6:** Multivariate predictors death in HIV-infected individuals at Jimma University Specialized Hospital, January 2006 to December 2011

Variable	Number of death	HR [95 % CI]	p value
Age			
Below 30	21	1	
30–39	11	0.898 [0.357, 2.261]	0.820
40–49	6	1.047 [0.317, 3.459]	0.940
Above 50	4	4.783 [1.076, 21.268]	0.040
BMI			
<18.5	25	1.460 [0.648, 3.287]	0.361
≥18.5	17	1	
Marital status			
Single	7	1	
Married	16	0.470 [0.173, 1.278]	0.139
Widowed	1	0.258 [0.028, 2.374]	0.232
Divorced	8	0.520 [0.154, 1.763]	0.294
CD4 count			
<100	20	1	
100–199	15	0.522 [0.217, 1.254]	0.146
>200	7	0.287 [0.097, 0.848]	0.02
Baseline WHO stage			
Stage I	6	1	
Stage II	9	1.540 [0.446, 5.315]	0.495
Stage III	17	1.501 [0.487, 4.631]	0.480
Stage IV	10	2.802 [0.657, 11.942]	0.164
Occupation			
Gov’t employee	14	3.608 [0.720, 18.090]	0.119
Unemployed	25	3.481 [0.740, 16.371]	0.114
Others	1	1	
Educational level			
Not educated	4	1	
Primary	17	2.293 [0.686, 7.667]	0.178
Secondary	14	2.376 [0.079, 8.317]	0.176
Tertiary	7	2.401 [0.494, 11.661]	0.277
Fluconazole prophylaxis			
No	34	1	
Yes	8	5.639 [1.811, 17.563]	0.003
Baseline HAART			
Stavudine based	19	1	
Zidovudine based	12	4.182 [1.550, 11.286]	0.005
Tenofovir based	11	5.156 [1.924, 13.819]	0.001

### Discussion

There was no significant difference between malnourished and well-nourished patients in terms of CD4 recovery after HAART initiation. This finding is similar with reports by Elizabeth et al. who reported nutritional status at the start of HAART as measured by BMI, FFMI, FMI and skin folds did not predict good post HAART CD4 change at 6, 12 and 24 months in a cohort of HIV infected Rwanda women [12]. There was no significant difference in CD4 recovery between malnourished patients and well-nourished patients after initiation of HAART [12]. Likewise, Brandon et al. showed that baseline BMI does not predict CD4 change at 48 weeks (11 months) but that this baseline BMI did predict CD4 change at 96 and 144 weeks [13]. Lack of longterm outcomes related to baseline BMI in our study may be related to different ethnic background, geographical region, diet, smaller sample size and shorter follow-up period.

CD4 count at the initiation of treatment was significantly associated with a change in CD4 at 6, and 12 months when adjusted for other variables. A CD4 count greater than 350 cells/mm^3^ at baseline was associated with smaller increase in CD4 at 6 and 12 months in multivariable model compared to patients with lower CD4 counts. This finding was also documented in London, UK where lower CD4 count was associated with greater increase in CD4 after 3 month of HAART initiation both in multivariable model [14]. In both our study and Bennett and his colleagues, females were found to have better CD4 recovery compared to males after at least a year of treatment [15]. Finally, we found that younger patients had greater increase in CD4 at 6, 12, and 24 months similar to study in Europe which shows younger age favors CD4 cell restoration [16] and increasing age was risk factors for not achieving CD4 count >200 [17]. Similarly, this finding is in line with the finding from study done in seven African countries which shows older age at HAART initiation was associated with suboptimal CD4 recovery CD4 [18]. Older age was independent predictor of mortality, which was comparable with study done in Johannesburg, South Africa which shows older patients were at greater risk of early death compared to younger patients [19]. Moreover, this finding was in line with study from South Eastern Nigeria and with what Adena et al. reported which shows patients whose age greater than 45 were at greater risk of early death [20].

Malnourished patients were almost two times more likely to die early compared to well-nourished group though it is not significant in Multivariable Cox proportional model. This finding was in line with study done in Zewditu Memorial hospital, Ethiopia which shows patients with BMI of <18.5 kg/m^2^ at the start of treatment were 1.13 [95 % CI 0.23, 5.43] times at greater risk of death compared to those with BMI >18.5 kg/m^2^ [21]. This finding was also consistent with the finding from study done in Singapore where patients with BMI less than 18.5 were 1.4 times more likely to die early compared to patients with BMI greater than or equal to 18.5 kg/m^2^ [12]. Studies in Tanzania and Malawi where malnourished patients were associated with early death compared to well-nourished group [22, 23]. Likewise, Raoul and his colleagues reported low BMI was an independent predictor of death in West Africa [18]. Other studies [13–15] also reported BMI as an important predictor of death.

Tenofovir based regimen was associated with highest risk of death compared to Stavudine and Zidovudine based regimen. This is similar with what Kelechi and his colleagues reported [24] but in contrast to the finding South Africa which shows there was no difference in mortality between Tenofovir and Stavudine [19]. Again different ethnic background, geographical region, diet, smaller sample size and shorter follow-up period in our study. Furthermore, single antiretroviral drugs were compared in our study while Kavindhran et al. [19] compared regimens of three or more antiretroviral drugs.

Independent of specific antiretrovirals, Fluconazole prophylaxis was found to be strong predictor of death in our study and indifference to a Uganda study suggesting that this antifungal did not impact mortality under similar conditions [25]. Most patients in our study took fluconazole when CD4 cell counts were <200/ml.

## Conclusion

BMI at the start did not predict change in CD4 at any time point after initiation of HAART after adjustment for other variables.

Age of the patients was significant predictor of immunologic outcome at 6, 12, 24 months adjusting for other factors. Baseline CD4 count was significant predictor of CD4 change at 12 months. Sex was significant predictor of immunologic outcome at 24 months after HAART initiation. At 24th month baseline HAART and marital status predicts immunologic outcome.

Age >50 years of age, Tenofovir based regimen, Zidovudine based regimen, taking fluconazole and CD4 <200 were associated with greater risk of death.

Malnutrition at the start of HAART does not predict early death of patients.

## References

[1] Palella FJ, Delaney KM, Moorman AC (1998). Declining morbidity and mortality among patients with advanced human immunodeficiency virus infection. Lancet.

[2] Valdez H, Chowdhry TK, Asaad R (2001). Changing spectrum of mortality due to human immunodeficiency virus: analysis of 260 deaths during 1995–1999. Clin Infect Dis.

[3] Floridia M, Fragola V, Galluzzo CM (2002). HIV-related morbidity and mortality in patients starting protease inhibitors in very advanced HIV disease (CD4 count of <50 cells/microL): an analysis of 338 clinical events from a randomized clinical trial. HIV Med.

[4] Feldman JG (2000). Serum albumin as a predictor of survival in HIV infected women in the women’s interagency HIV study. AIDS..

[5] Feldman JG (2003). Serum albumin is a powerful predictor of survival among HIV-1-infected women. J Acquir Immune Defic Syndr..

[6] Degu Jerene AE, Yewubnesh H, Lindtjørn Bernt (2006). Predictors of early death in a cohort of Ethiopian patients treated with HAART. BMC Infect Dis.

[7] Asgeir Johannessen EN, Bernard JN, Leiv S (2008). Predictors of mortality in HIV-infected patients starting antiretroviral therapy in a rural hospital in Tanzania. BMC Infect Dis.

[8] Tang AMFJ, Spiegelman D, Knox TA (2002). Weight loss and survival in HIV-positive patients in the era of highly active antiretroviral therapy. J Acquir Immune Defic Syndr.

[9] Paton NI, Earnest A, Bellamy R (2006). The impact of malnutrition on survival and the CD4 count response in HIV-infected patients starting antiretroviral therapy. HIV Med..

[10] Elizabeth DR (2011). Association of pre-treatment nutritional status with change in CD4 count after antiretroviral therapy at 6, 12, and 24 months in Rwandan women. PLoS ONE.

[11] University Specialized hospital. http://www.ju.edu.et/Jimma 2014. Accessed 23 Sep 2014.

[12] Brandon RJ (2011). Body mass index and CD4+ T-lymphocyte recovery in HIV infected men with viral suppression on antiretroviral therapy. HIV Clin Trials..

[13] Colette J (2004). Factors influencing increases in CD4 cell counts of HIV-positive persons receiving long-term highly active antiretroviral therapy. J Infect Dis.

[14] Bennett KK (2002). Baseline predictors of CD4 T-lymphocyte recovery with combination antiretroviral therapy. J Acquir Immune Defic Syndr.

[15] Jean-paul AM (2001). Infulence of age on CD4 cell recovery in human immunodeficiency virus-infected patient’s receiving highly active antiretroviral therapy: evidence from the EuroSIDA study. J Infect Dis.

[16] Engsig FN (2014). Long-term mortality in HIVpositive individuals virally suppressed for >3 years with incomplete CD4 recovery. Clin Infect Dis.

[17] Aida YZ (2014). Predictors of suboptimal CD4 response among women achieving virologic suppression in a randomized antiretroviral treatment trial, Africa. BMC Infect Dis.

[18] Desta GG (2013). Virologic and immunologic outcome of HAART in Human Immunodeficiency virus (HIV)-1 infected patients with and without tuberculosis (TB) and latent TB infection(LTBI) in Addis Ababa, Ethiopia. AIDS Res Ther..

[19] Maskew Mhairi (2011). Poorer ART outcomes with increasing age at a large public sector HIV Clinic in Johannesburg, South Africa. J Int Assoc Phys AIDS Care..

[20] Adena H (2008). Effect of age and HAART regimen on clinical response in an urban cohort of HIV infected individuals. AIDS..

[21] Zachariah RFM, Massaquoi M, Pasulani O (2006). Risk factors for high early mortality in patients on antiretroviral treatment in a rural district of Malawi. AIDS..

[22] Enju Liu DS, Semu Helen, Hawkins Claudia (2011). Nutritional status and mortality among HIV infected patients receiving antiretroviral therapy in Tanzania. J Infect Dis.

[23] Kelechi N, et al. Determinants of mortality among adult HIV-infected patients on antiretroviral therapy in a rural hospital in Southeastern Nigeria: a 5-year cohort study. AIDS Res Treat 2014;2014:1–6, Article ID 867827. doi:10.1155/2014/867827. 10.1155/2014/867827 PMC4140117 25165579

[24] Kavindhran JL (2013). Comparison of Tenofovir, Zidovudine, or Stavudine as part of first-line antiretroviral therapy in a resource limited-setting: a cohort study. PLoS ONE.

[25] Parkes-Ratanshi WK (2011). Primary prophylaxis of cryptococcal disease with fluconazole in HIV-positive Ugandan adults: a double-blind, randomised, placebo-controlled trial. Lancet Infect Dis..

